# The effect of workplace environment on coal miners' gut microbiota in a mouse model

**DOI:** 10.3389/fmicb.2024.1453798

**Published:** 2024-12-11

**Authors:** Lei Li, Mei Zhi, Siwei Wang, Jun Deng, Qing Cai, Dayun Feng

**Affiliations:** ^1^School of Safety Science and Engineering, Xi'an University of Science and Technology, Xi'an, China; ^2^Key Laboratory for Prevention and Control of Coal Fires in Shaanxi Province, Xi'an, China; ^3^Safety Supervision Department, Nanhai Department of Transportation, Foshan, China; ^4^Department of Neurosurgery and Institute for Functional Brain Disorders, Tangdu Hospital, Fourth Military Medical University, Xi'an, China

**Keywords:** coal mine workplace environment, intestinal dysfunction, gut microbiota, compound probiotics, intervention

## Abstract

The coal mine workplace environment is a significant factor in inducing occupational health issues, such as intestinal dysfunction in coal miners. However, the mechanism by which the coal mine workplace environment induces intestinal dysfunction is still unclear. Therefore, we applied the Coal Mine Workplace Environment Biological Simulation (CEBS) model which was previously constructed to detect the intestinal pathological manifestations and changes in the gut microbiota of mice from the perspectives of intestinal function, tissue morphology, and cell molecules. CEBS mice showed increased fecal water content, shortened colon length, significant activation of MPO^+^ and CD11b^+^ numbers, and significant changes in IL-1b, IL-6, and IL-12 expression levels. In addition, we also found an imbalance in the proportions of *Firmicutes, Bacteroidetes, Lactobacillus*, and *Parabacteroides* in CEBS mice, resulting in significant changes in gut microbial diversity. After intervention with compound probiotics, the intestinal function of CEBS + Mix mice was improved and inflammation levels were reduced. Results indicated that stress in the coal mine workplace environment can lead to intestinal dysfunction and inflammatory damage of the colon and use of compound probiotics can improve intestinal dysfunction in CBES mice. In our study, we revealed that there is a correlation between coal mine workplace environment and diversity disorders of gut microbiota. This discovery has enhanced the relevant theories on the causes of intestinal dysfunction in coal miners and has suggested a new approach to intervention.

## 1 Introduction

The underground workplace environment of coal mines is complex. Coal miners have been in a stressful environment composed of high temperature, high humidity, dust, and other factors for a long time (Lu et al., 2020; Xie et al., 2020; Yin et al., 2022), resulting in prominent occupational health problems such as cardiovascular and cerebrovascular diseases, central nervous system, and immune system diseases (Ijaz et al., 2020). Among them, the prevalence of intestinal dysfunction such as irritable bowel syndrome and functional dyspepsia is higher among coal miners compared to the social average (Sperber et al., 2017, 2021). This has become one of the significant factors that seriously impact the physical and mental health of coal miners. However, current research on intestinal dysfunction in coal miners is limited to epidemiological investigation, and the mechanism of linking coal mine workplace environment to intestinal dysfunction in coal miners is not yet understood. At the same time, how to effectively intervene in intestinal dysfunction in coal miners from a physiological perspective has become an urgent problem to be solved.

The causes of intestinal dysfunction are diverse and complex. In addition, a large number of clinical and basic research confirmed factors such as genetics (Whorwell et al., 1986; Levy et al., 2000; Kanazawa et al., 2004; Saito et al., 2010), environment (Arnesen et al., 2021; Yang et al., 2022), psychology (Whitehead et al., 2002; Kassinen et al., 2007; Wu, 2011), and diet (Adolph and Zhang, 2022; Seethaler et al., 2022). Gut microbiota has been found to play an increasingly important role in intestinal dysfunction (Barlow et al., 2021; Lyra et al., 2009). The gut microbiota maintains various physiological homeostasis of the host. This homeostasis will be disrupted when the number or structure of the gut microbiota changes. Leading to damage to the intestinal barrier and intestinal inflammation (Ni et al., 2017), thereby causing various diseases such as inflammatory bowel disease (Zahraa et al., 2020). Gut microbiota becomes a potential target for regulating neurological dysfunction. Studies have shown that there is an imbalance of gut microbiota in individuals with intestinal dysfunction. For example, compared to normal individuals, the relative abundance of *Firmicutes* decreases (Sokol et al., 2008; Zoetendal et al., 2008) and the relative abundance of *Proteobacteria* increases in IBD patients (Seksik et al., 2003; Baumgart et al., 2007). *Bacteroidetes, Firmicutes*, and *Proteobacteria* are the main bacteria that distinguish IBD patients from healthy individuals (Yilmaz et al., 2019). The relative abundance of *Enterobacteria* increases in UC patients (Frank et al., 2007). However, research on the gut microbiota of coal miners is relatively limited. The relative abundance of *Actinobacteria, Bacteroideales*, and *Bifidobacteriaceae* increased among ordinary underground coal miners (Lu et al., 2021). Coal miners with pneumoconiosis have seen a decrease in the *Microporaceae, Actinobacteria*, and *Bifidobacteria*, while the *Prevotellaceae* has increased (Li Y. D. et al., 2022).

The gut microbiota has a dual effect on host health, and the disruption of gut microbiota community structure may lead to the occurrence of diseases such as intestinal and neurological disorders (Kelly et al., 2016; Park et al., 2013). Treatment can also be achieved by supplementing probiotics to regulate gut microbiota homeostasis. Fecal microbiota transplantation (FMT), antibiotics, single or multiple probiotic combination interventions, and other methods for regulating intestinal microbiota have been widely used in clinical and preclinical settings (Liu et al., 2022; Mangiola et al., 2016). FMT extracts and supplements the gut microbiota from the feces of healthy individuals to patients, and its therapeutic effect is greatly influenced by the microbiota of the donor (Paz et al., 2024; Shalini et al., 2022). Probiotics can stably colonize the intestine and regulate the composition of gut microbiota (Maldonado-Gómez et al., 2016). It plays an important role in the prevention and treatment of acute gastritis (Jafarnejad et al., 2016). Therefore, there is a direct correlation between probiotic levels and intestinal health. Some studies have compared the use of several probiotics in a combination group to treat inflammatory bowel disease model mice with a single strain treatment group. The probiotic combination group showed more significant therapeutic effects (Wang et al., 2022; Xu et al., 2022).

In this work, the CEBS device which was previously constructed by Li L. et al. (2022) used to clarify the relationship between coal mine workplace environment and intestinal dysfunction at the physiological level. Our goal is to explore the changes in intestinal function, pathological characteristics of intestinal tissue, and intestinal microbial community under the stress in coal mine workplace environment are explored. Based on this, compound probiotics has been used to intervene in model mice to explore potential means of improving intestinal dysfunction in coal miners. This provides a theoretical reference for revealing the pathogenesis of intestinal dysfunction and improving the physical and mental health of coal miners.

## 2 Materials and methods

### 2.1 Animals and experimental design

**Figure 1:**
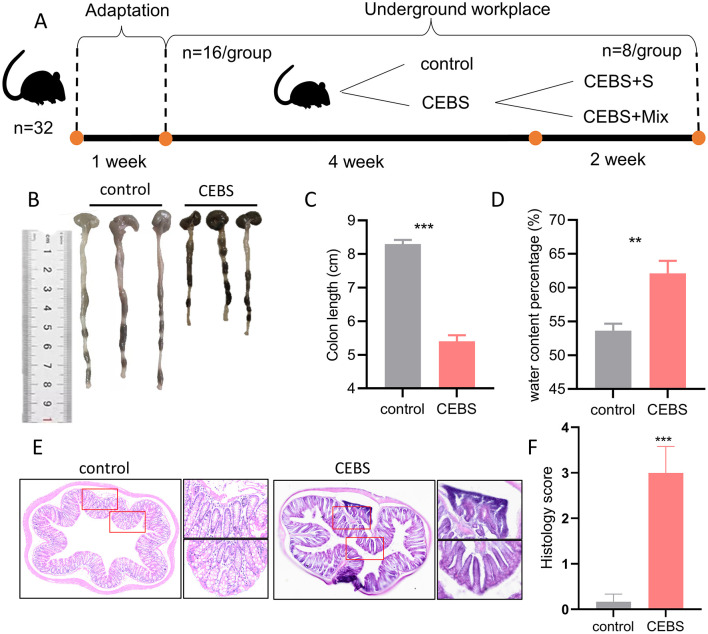
CEBS mice show colon function damage. **(A)** The experimental procedures of this study. **(B)** Macroscopic view of colon tissue. **(C)** Colon tissue length. **(D)** Fecal water content percentage. **(E)** H&E staining in colon tissue. **(F)** Histology score (control mice *vs*. CEBS mice). Scale = 250 μm; **P < 0.001, and ***P < 0.001.

Male C57BL/6 mice aged 8 weeks (20–22 g) were charged from the Experimental Animal Center of the Fourth Military Medical University. All experimental procedures were approved by the Animal Use and Care Committee of the University. All the mice (*n* = 32) had 1 week of environmental adaptation after purchase, then were randomly divided into two groups (*n* = 16 per group): (1) control group, (2) CEBS group. The CEBS model procedure has been reported in our previous research. Briefly, the CEBS model was implemented as follows: the finely divided coal cinder with a thickness of 2–3 cm in the cages [15 cm (L) ^*^ 30 cm (W) ^*^ 15 cm (H)] was placed as bedding to simulate the ground conditions of the mine. The temperature (20 ± 2°C), noise (1,000 Hz, 75 dB), humidity (70% ± 5%) and light (80 Lux) parameters are set in accordance with the standards of the Coal Mine Safety Regulations. Toxic gases such as CO, H_2_S, SO_2_ and Methane have not been added to the current model because they are present at very low levels in coal mines that meet safety standards (Methane: 0–1%, CO: 0%−0.0024%, H_2_S: 0–0.005%, SO_2_: 0–0.00025%). The mice entered the simulated environment at 9 a.m. and then returned to the breeding cage at 5 p.m. according to the workplace hours of coal miners. There was no food and water supply during the workplace period. The control group was used to verify the stress effect of coal mine workplace environment on coal miners. The mice in the control group were acclimatized (12-h light/dark cycle) under standard conditions with freedom access to food and water. The mice were immediately sacrificed to collect brain, colon, and fecal matter after 4 weeks simulation. The CEBS group was divided into two groups (*n* = 8 per group): (1) CEBS + S group, (2) CEBS + Mix group. From the 4th week, the mice in the CEBS + Mix group were given compound probiotics, while the CEBS + S group were given normal saline. After 2 weeks of intervention, the result of compound probiotics on intestinal pathology was analyzed (Figure 1A).

### 2.2 Fecal excretion experiment

After a 2-h fasting period, the mice were moved into a clean transparent plastic cage for an additional 2 h. Fecal particles were collected and counted, and the fresh fecal wet weight was recorded. Subsequently, the dry weight of the feces was measured after drying at 85°C for 24 h. Fecal water content (%) was calculated using the formula: (wet weight – dry weight)/wet weight ^*^ 100%. The fecal water content of CEBS and control mice was measured during the final week of adaptation to the coal mine workplace environment over seven consecutive days. In the last week of modeling, the fecal water content of CEBS + S and CEBS + Mix mice was measured for seven consecutive days. Finally, the mean values were compared to determine the fecal water content of the mice.

### 2.3 Hematoxylin and eosin staining

The colon tissues were collected and fixed in 4% paraformaldehyde overnight and then embedded with paraffin. The tissues were sectioned by a slicer (5 μm) and stained with hematoxylin and eosin (H&E) according to the standard H&E protocol (Pan et al., 2020).

### 2.4 Immunofluorescence staining

Immunofluorescence was performed as previously described. Mice were deeply anesthetized by inhalation of isoflurane and perfused transcranial with 20 ml 0.9% phosphate buffered saline (PBS) followed by 30 ml 4% PFA. Brains and colons were post-fixed in 4% PFA at 4°C overnight, then cryoprotected in 30% sucrose 0.1 M PBS over 3 days. Coronal slices (20 μm) were obtained through the frozen slicer. Slices were blocked with 3% fetal bovine serum, 0.3% Triton X-100 0.01 M PBS for 2 h at room temperature and then incubated in primary antibodies. DAPI staining solution was added dropwise, and incubated at room temperature for 15 min, and then an anti-quenching fluorescent mounting agent was added dropwise for sealing. Observe under a confocal microscope and acquire images.

### 2.5 16S rRNA gene sequencing

The 16S rRNA gene sequencing extracts the DNA of the microbial flora, targeting a specific section of the variable region (V3–V4) for PCR amplification, and then analyzing the gene composition and function of the microbial population in a specific environment using high-throughput sequencing. This helps in studying microbial diversity and abundance and then analyzing the relationship between microorganisms and the environment, including hosts. The sequencing experiment process mainly includes sample DNA extraction, PCR amplification of designated regions, library preparation, quality inspection and quantification, sequencing, and bioinformatics analysis.

Frozen mice fecal samples were collected in sterile EP tubes and stored at 80°C for further analysis. Microbial DNA was extracted from fecal samples using the QIAamp^®^ Fecal Fast DNA Stool Mini Kit (Qiagen, Germany). The V3-V4 hypervariable region of the 16S rRNA gene was amplified using PCR with primers (F: 5′-ACTCCTACGGGAGGCAGCA-3′, R: 5′-GGACTACHVGGGTWTCTAAT-3′). PCR products were token for constructing libraries and sequenced on an Illumina HiSeq PE250 platform (Illumina, USA) at Personalbio (Shanghai, China). Subsequently, the QIIME2 platform was used to perform microbiome sequence noise reduction on the sequencing data (Bolyen et al., 2019).

### 2.6 Statistical analysis

*T*-test or one-way analysis of variance was used to test the differences between different groups. Data conforming to normal distribution were expressed as mean ± standard error of the mean (SEM). The Kruskal–Wallis *H*-test is used to analyze the significance of data with non-normal distribution, and the data are represented by the InterQuqrtile Range (IQR). Immunofluorescence images were analyzed by Image J software for cell counting and mean fluorescence intensity, and SPSS 26.0 software was used for statistical analysis. GAPDH was used as an internal reference gene, and the relative expression level of mRNA was calculated by 2^−ΔΔCT^ method. The *P*-values (both for amplicon-sequencing and qPCR data) were adjusted with Benjamini–Hochberg approach for false discovery rate (FDR), and an adjusted *P*-value < 0.05 indicates statistical significance.

## 3 Results

### 3.1 Coal mine workplace environment induced colon tissue damage in CEBS mice

In order to explore the effect of coal mine workplace environment on the occurrence of intestinal diseases in mice, the colon symptoms of mice were measured after 4 weeks of adaptation to the coal mine workplace environment. Compared with the control mice, the CEBS mice showed a significantly decreased of colon length [Figures 1B, C, 95% CI, (−3.381, −2.419); *P* < 0.001]. It may result in a reduced ability to absorb water and minerals. And the fecal water content percentage was significantly increased [Figure 1D, 95% CI, (3.833, 13.120); *P* < 0.01]. CEBS mice exhibit diarrhea symptoms. Subsequently, to further analyze the extent of colonic injury in CEBS mice, colon tissues were stained with H&E and histology analysis. Histology score is usually used to evaluate colitis in animal models. The score is derived from the observation and quantification of specific pathological features of intestinal tissue, including inflammatory cell infiltration, destruction of crypt structures, and epithelial cell injury, etc. Using a 0–4 scoring system, a score of 0 indicates normal tissue, while a score of 4 indicates the most severe lesions (Jang et al., 2019). Compared with the control group, the inflammatory response in the colon tissue of CEBS mice exhibited severe infiltration of basal inflammatory cells, atrophy of epithelial crypts, increase of crypt spacing, and decrease of crypt number (Figure 1E). The histology score of colon tissue in CEBS mice was significantly higher than control mice [Figure 1F, 95% CI, (−4.042, −1.624); *P* < 0.001]. These results indicated that the colon tissue was damaged but no obvious epithelial barrier damage in CEBS mice, which may be due to the stress in coal mine workplace environment.

### 3.2 Coal mine workplace environment promoted the inflammatory response of colon tissue in CEBS mice

**Figure 2:**
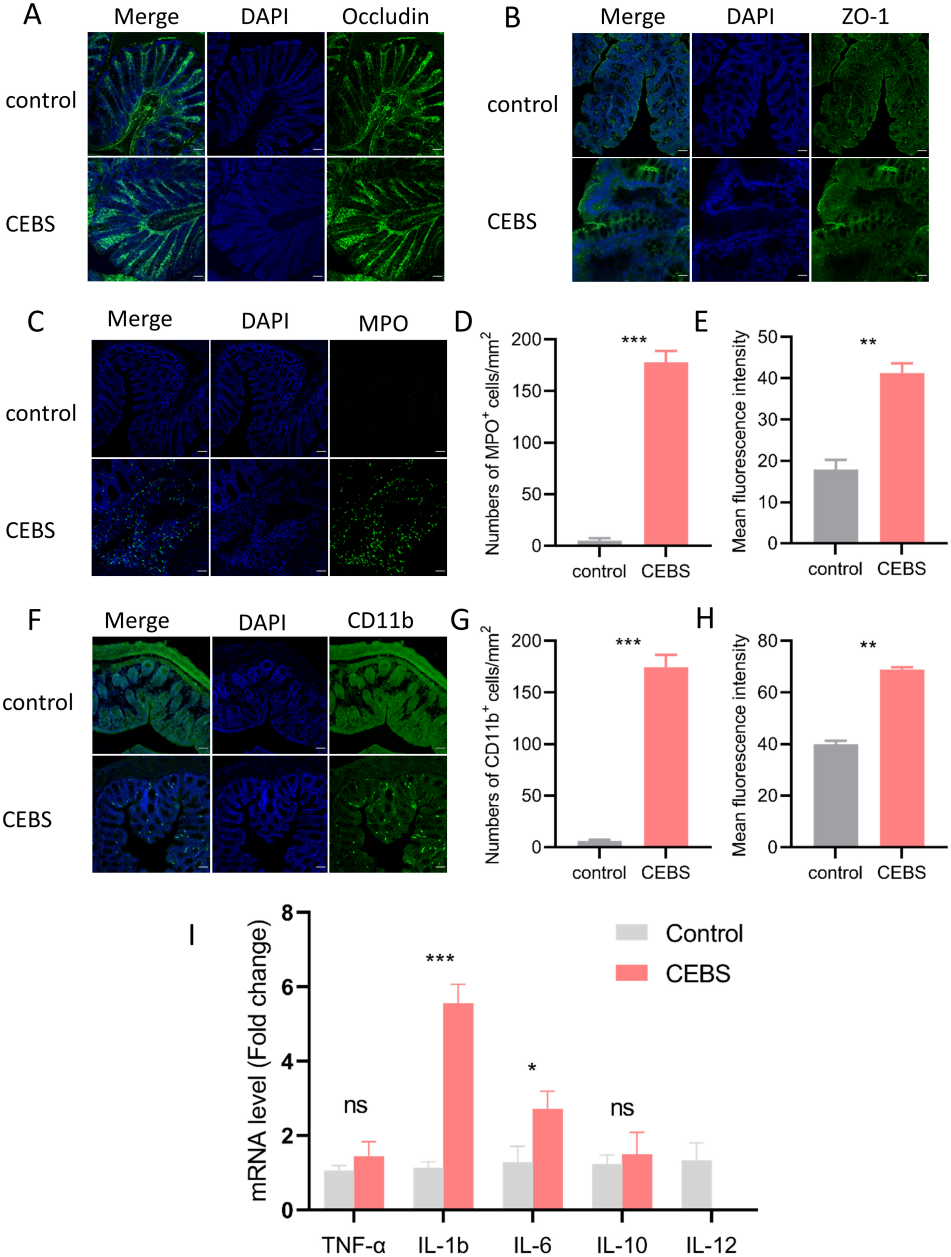
CEBS mice show severe colonic inflammatory injury. **(A)** Immunofluorescence staining of Occludin. **(B)** Immunofluorescence staining of ZO-1. **(C)** Immunofluorescence staining of MPO+ cells. **(D)** Numbers of MPO+ cells. **(E)** Mean fluorescence intensity of MPO+ cells. **(F)** Immunofluorescence staining of CD11b+ cells. **(G)** Numbers of CD11b+ cells. **(H)** Mean fluorescence intensity of CD11b+ cells. **(I)** The mRNA expression level[[Inline Image]] of inflammatory factors in colon tissue (control mice vs. CEBS mice). Scale = 50 μm; ns, *P* > 0.05, *P < 0.05, **P < 0.001, and ***P < 0.001.

To futher research the influence of coal mine workplace environment on colon inflammatory, immunofluorescence and qRT-PCR were performed to detect inflammation. We evaluated barrier-related proteins in CEBS mice colon tissues by immunofluorescence. Consistent with the results of H&E staining, the expressions of Occludin and ZO-1 in CEBS mice colon tissues were not decreased compared with those of control mice (Figures 2A, B). In present study, we performed immunofluorescence staining of the colon to detect the activation of immune cells, using MPO^+^ as the marker of neutrophils, and CD11b^+^ as the marker of macrophages. Compared with control mice, MPO^+^ cells (Figure 2C) and CD11b^+^ cells (Figure 2F) were significantly elevated in the colon tissues of CEBS mice. The numbers of MPO^+^ cells [Figure 2D, 95% CI, (147.3, 198.1); *P* < 0.0001] and CD11b^+^ cells [Figure 2G, 95% CI, (141.0, 195.5); *P* < 0.0001] were significantly increased, and the mean fluorescence intensity of MPO^+^ cells [Figure 2E, 95% CI, (9.773, 37.020); *P* < 0.001] and CD11b^+^ cells [Figure 2H, 95% CI, (25.86, 31.92); *P* < 0.001] also had significant difference between control mice and CEBS mice. Further, we measured the production of inflammatory mediators associated with macrophages and neutrophils. As shown in Figure 2I, there was no significant difference in TNF-α and IL-10 levels between the two groups. Proinflammatory cytokines IL-1b and IL-6 in CEBS mice were markedly up-regulated [IL-1b, 95% CI, (−5.993, −2.875); *P* < 0.0001; IL-6, 95% CI, (−3.029, 0.161); *P* < 0.05], and the anti-inflammatory cytokine IL-12 was markedly down-regulated [95% CI, (−0.834, 1.821); *P* < 0.05]. The result suggested that the development of colonic inflammation in CEBS mice may lead to pathological changes in colonic function. Collectively, these results indicated that coal mine workplace environment could significantly stimulate the inflammatory response of colon tissue.

### 3.3 Coal mine workplace environment changed the diversity of gut microbiota in CEBS mice

**Figure 3:**
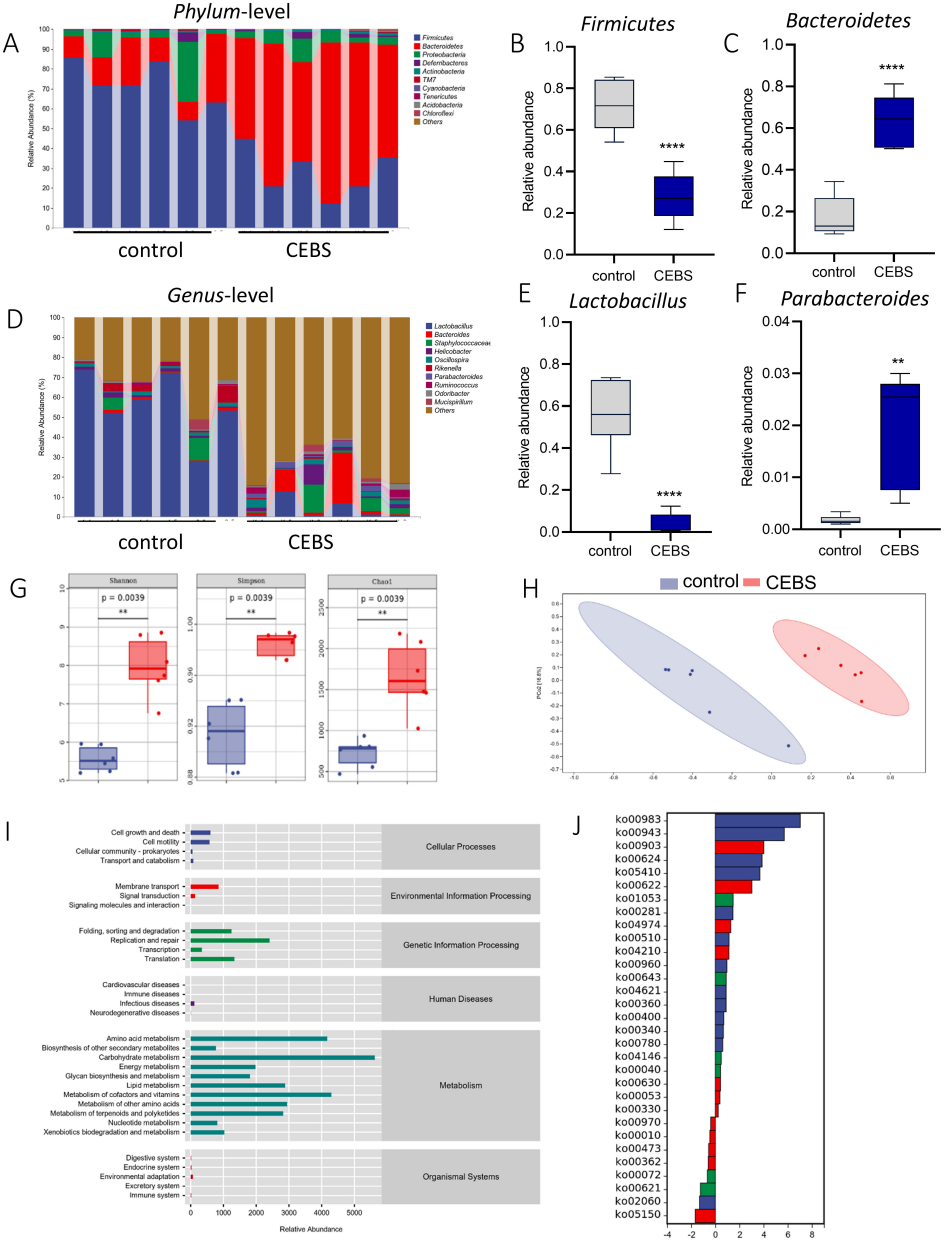
CEBS mice show gut microbiota disorder. **(A)** The relative abundance of gut microbiota at *Phylum*-level. **(B)** The relative abundance of *Firmicutes*. **(C)** The relative abundance of *Bacteroidetes*. **(D)** The relative abundance of gut microbiota at *Genus*-level. **(E)** The relative abundance of *Lactobacillus*. **(F)** The relative abundance of *Parabacteroides*. **(G)** Alpha diversity analysis (Chao, Shannon, Simpson). **(H)** PCoA of gut microbiota. **(I)** Pathway statistics of metabolic. **(J)** Differential metabolic pathway statistics of metabolic (control mice *vs*. CEBS mice). **P < 0.01, and ****P < 0.0001.

Disturbance of gut microbiota structure is an important cause of intestinal dysfunction. We used 16S rRNA sequencing to analyze the diversity of gut microbiota in CEBS mice and its impact on intestinal function. Sequencing of gut microbiota DNA fragments from 12 fecal samples (Table 1) resulted in an average sequence length between 400–450 bp, which meets the sequencing requirements. Randomly select 95% of the lowest sample sequence from each sample to achieve a unified sequencing depth, ensuring the accuracy of subsequent data analysis (Supplementary Figure S1A). Using the Greengenes database for sequence comparison, the taxonomic annotation results showed a significant increase in gut microbiota types in CEBS mice after exposure to coal mine workplace environment (Table 2; Supplementary Figure S1B). We compared the relative abundances of microbes at various taxon (Table 3; *Class*-level, Supplementary Figure S2A; *Family*-level, Supplementary Figure S2D; *Species*-level, Supplementary Figure S2K; *Phylum*-level, Figure 3A; *Genus-*level, Figure 3D). At the *Phylum*-level, in CEBS mice compared with control mice, the relative abundance of *Firmicutes* showed significant decrease [Figure 3B, 95% CI, (−0.5916, −0.2837); *P* < 0.001], *Bacteroidetes* showed significant increase [Figure 3C, 95% CI, (0.3164, 0.6126); *P* < 0.001], and no significant change in *Proteobacteria* and *Actinobacteria* (Supplementary Figures S2B, C, ns). At the *Genus*-level, the relative abundance of *Lactobacillus* [Figure 3E, 95% CI, (−0.6833, −0.3672); *P* < 0.0001] showed significant decrease, *Parabacteroides* showed significant increase [Figure 3F, 95% CI, (0.00866, 002827); *P* < 0.01], and no significant change in *Rikenella* and *Bacteroides* (Supplementary Figures S2E, F). At the *Family*-level, there were also significant changes in the relative abundance of gut microbiota (Supplementary Figures S2G–J). The above results indicated that the gut microbiome community structures in mice had been hugely changed by the coal mine workplace environment.

**Table 1:** Quality statistics of intestinal microbial sequencing.

**Groups**	**Simples**	**Input**	**Filtered**	**Denoised**	**Merged**
Control	1	43,738	39,485	38,335	35,480
	2	46,494	42,789	42,352	41,772
	3	46,975	43,130	42,185	39,969
	4	43,223	38,854	37,977	35,864
	5	44,189	40,257	39,137	36,824
	6	51,670	46,362	44,868	42,002
CEBS	1	44,641	40,155	38,834	34,633
	2	46,997	42,712	41,617	38,385
	3	50,074	45,594	44,061	39,034
	4	44,150	39,716	38,869	36,751
	5	49,517	43,990	42,313	38,259
	6	49,386	44,823	42,568	36,175

**Table 2:** Statistics of the number of classification units.

**Groups**	**Simples**	**Domain**	**Phylum**	**Class**	**Order**	**Family**	**Genus**	**Species**
Control	1	1	8	15	18	29	32	16
	2	1	8	16	22	39	38	17
	3	1	10	17	22	33	33	21
	4	1	9	20	29	41	38	18
	5	1	8	15	19	37	44	24
	6	1	8	15	18	27	27	12
CEBS	1	1	8	16	19	34	38	15
	2	1	6	14	20	33	40	29
	3	1	10	20	22	38	47	18
	4	1	8	15	19	34	42	27
	5	1	15	37	45	55	51	25
	6	1	19	46	57	71	63	27

**Table 3:** Relative abundance of gut microbiomes at different taxonomic levels.

**Taxonomic level**	**Relative abundance**		***P*-value**
	**Control**	**CEBS**	
**Phylum**			
*Firmicutes*	0.6994	0.2784	0.000
*Bacteroidetes*	0.2443	0.6388	0.001
*Proteobacteria*	0.0399	0.0559	0.480
*Actinobacteria*	0.0088	0.0093	0.839
**Family**			
*Lactobacillaceae*	0.5613	0.1727	0.000
*Rikenellaceae*	0.0979	0.0149	0.091
*Lachnospiraceae*	0.0125	0.0759	0.062
*S24-7*	0.1141	0.4979	0.000
*Porphyrommo*	0.0029	0.0204	0.003
**Genus**			
*Lactobacillus*	0.5603	0.0375	0.000
*Bacteroides*	0.0108	0.0688	0.180
*Rikenella*	0.0662	0.0040	0.108
*Staphylococcus*	0.0111	0.0436	0.208
*Parabacteroides*	0.0029	0.0202	0.003

Analysis of α-diversity were used to evaluated the richness and diversity of bacterial species. The Shannon [95% CI, (1.632, 3.193)], Simpson [95% CI, (0.04569, 0.09595)], Chao1 [95% CI, (939.3, 1163.7); Figure 3G, *P* = 0.0039], and other indexes (Supplementary Figures S3A–D) were significantly increased in CEBS mice compared to control mice. Analysis of β-diversity showed the gut microbiota composition of CEBS mice and control mice was obviously divided into two clusters based on weighted_unifrac (Figure 3H). ASV abundance was further used to analyze common ASVs sets, only 646 ASVs sets were common to both groups (Supplementary Figure S2C). The gut microbiota gene families were mapped to the KEGG database, and the MinPath algorithm was used to infer the existence of metabolic pathways, and the abundance data of metabolic pathways in each sample were obtained. As shows in Figure 3I, the acquired metabolic pathways were mainly enriched in the categories of Metabolism and Genetic Information Processing. The differential analysis of enriched metabolic pathways was performed (Figure 3J). A total of 31 significantly different metabolic pathways were obtained, including 23 pathways with significantly increased metabolic levels. The results indicated that the coal mine workplace environment may cause gut microbiota metabolic disorders and colonic inflammation.

### 3.4 Compound probiotics intervention reduced colon tissue damage in CEBS + Mix mice

**Figure 4:**
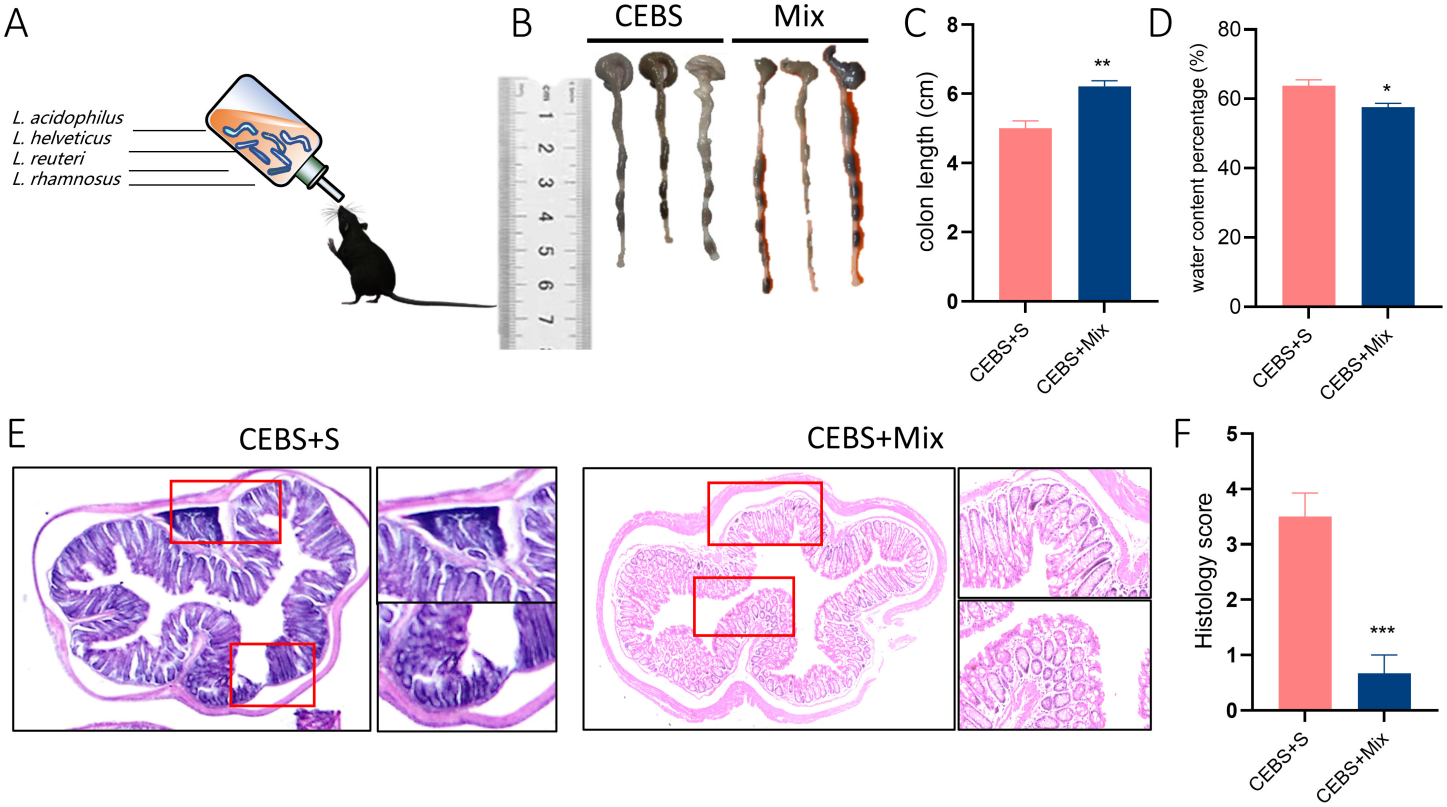
CEBS + Mix mice improved in colon tissue damage. **(A)** Schematic diagram compound probiotics intervention. **(B)** Macroscopic view of colon tissue. **(C)** Colon tissue length. **(D)** Fecal water content percentage. **(E)** H&E staining in colon tissue. **(F)** Histology score (CEBS + S mice *vs*. CEBS + Mix mice). Scale = 250 μm; *P < 0.05, **P < 0.01, and ***P < 0.001.

In order to alleviate colonic diseases caused by coal mine workplace environment. Based on the structural changes of gut microbiota in CEBS mice (Supplementary Figure S2D). *Lactobacillus Royale* (L.PB-LR09), *Lactobacillus suisse* (PB-LR76), *Lactobacillus rhamnosus* (HH-LA26), and *Lactobacillus acidophilus* (HH-LH17) were mixed for compound probiotics. CEBS + Mix mice were treated with compound probiotics and CEBS+S mice were intervened with saline for two-week intervention (Figure 4A). As excepted, compound probiotics treatment significantly alleviated colonic disease in CEBS mice, increased colon length [Figures 4B, C, 95% CI, (0.6149, 0.8180); *P* < 0.01], and decreased fecal water content in CEBS + Mix mice [Figure 4D, 95% CI, (−6.223, −1.632]; *P* < 0.05]. It showed that the compound probiotics intervention improved intestinal digestion in CEBS mice. To further verify the improvement, H&E staining was performed on the colon tissues of CEBS + Mix mice. The colon tissues of CEBS + S mice still showed significant pathological features such as inflammatory cell infiltration and crypt atrophy, whereas the crypt structure of CEBS + Mix mice showed intact crypt structure, increased crypt number, and the presence of only a small amount of inflammatory cell infiltration (Figure 4E), and the histology scores were reduced [Figure 4F, 95% CI, (−4.042, −1.624); *P* < 0.001]. It indicated that the damage to colon tissues caused by stress in coal mine workplace environment was significantly improved after the compound probiotics intervention.

### 3.5 Compound probiotics intervention reduced the inflammatory response of colon tissue in CEBS + Mix mice

**Figure 5:**
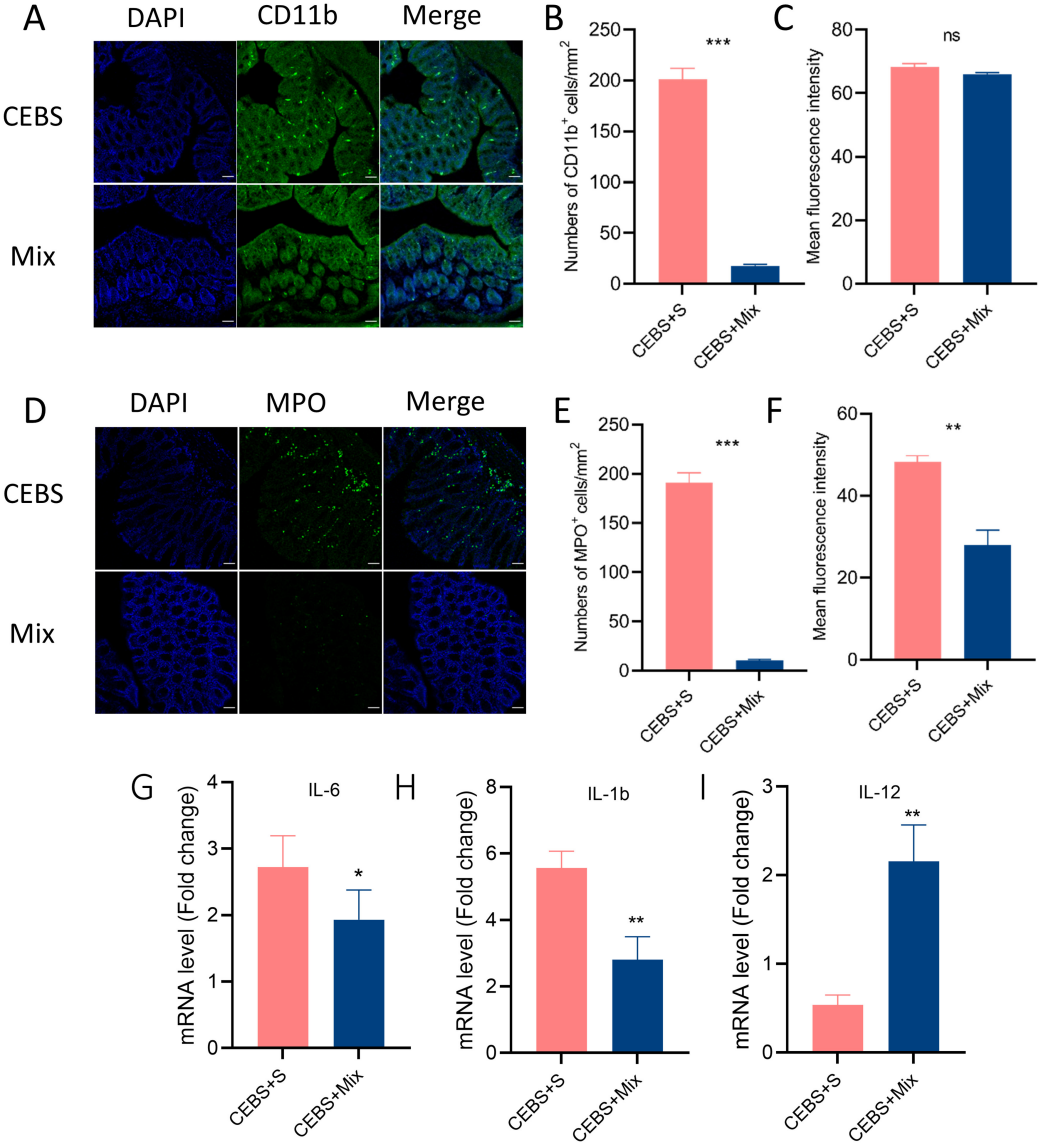
CEBS + mix mice improved in colonic inflammation. **(A)** Immunofluorescence staining of CD11b^+^ cells. **(B)** Numbers of CD11b^+^ cells. **(C)** Mean fluorescence intensity of CD11b^+^ cells. **(D)** Immunofluorescence staining of MPO^+^ cells. **(E)** Numbers of MPO^+^ cells. **(F)** Mean fluorescence intensity of MPO^+^ cells. **(G)** The mRNA level of IL-6. **(H)** The mRNA level of IL-1b. **(I)** The mRNA level of IL-12 (CEBS + S mice *vs*. CEBS + Mix mice). ns, *P* > 0.05, *P < 0.05, **P < 0.01, and ***P < 0.001.

Immunofluorescence staining was used to detect the inflammatory responses of MPO^+^ neutrophils and CD11b^+^ macrophages in colon tissue after intervention. The results showed that after 2 weeks of compound probiotics intervention, the number of CD11b^+^ macrophages aggregated into colon tissue was significantly decreased [Figures 5A, B, 95% CI, (−208.3, −159.0); *P* < 0.001. Figure 5C, 95% CI, (−5.284, 1.108); *P* > 0.05], and MPO^+^ also showed decreased [Figures 5D, E, 95% CI, (−180.9, −159.3); *P* < 0.001. Figure 5F, 95% CI, (−20.32, −13.84); *P* < 0.05]. The stress in the coal mine workplace environment led to a disturbance in the expression levels of pro-inflammatory and anti-inflammatory cytokines. CEBS mice showed an increase in pro-inflammatory cytokines IL-6 and IL-1b, while anti-inflammatory cytokines IL-12 decreased. After compound probiotics intervention, the results were consistent with immunofluorescence staining. Compared with CEBS + S mice, the expression of pro-inflammatory cytokine IL-6 [Figure 5G, 95% CI, (−2.244, 0.654); *P* < 0.05] and IL-1b was decreased [Figure 5H, 95% CI, (−4.672, −0.845); *P* < 0.01] and the expression of anti-inflammatory cytokine IL-12 was increased in colon tissues of CEBS + Mix mice [Figure 5I, 95% CI, (0.556, 2.683); *P* < 0.05]. Meanwhile, our previous research found that the coal mine workplace environment can induce anxiety in mice, while the compound probiotics treatment alleviated anxiety-like emotional behaviors.

## 4 Discussion

The previous results showed that the constructed CEBS model was able to respond well to the stress in coal mine workplace environmental. To further investigate the relationship between coal mine workplace environment and intestinal dysfunction, we studied the intestinal pathological manifestations of CEBS mice at the intestinal function, tissue morphology, and cellular and molecular levels. After adapting to a simulated coal mine workplace environment for 4 weeks, the CEBS mice displayed significant inflammatory responses. This included shortened colon length, increased fecal water content, severe infiltration of inflammatory cells, atrophy of epithelial crypts, increased crypt spacing, and decreased number of crypts, all of which were associated with diarrhea and other intestinal symptoms. These findings suggest that the coal mine workplace environment can lead to pathological changes in intestinal function.

Intestinal barrier and intestinal inflammation are the two most prevalent aspects of intestinal pathological manifestations (Black et al., 2020; Black and Ford, 2020). Studies have shown that the occurrence of UC is closely related to the disruption of intestinal barrier and gut microbiota (Loftus, 2004; Park and Jeen, 2018). To analyze the pathological changes of the intestinal tract caused by the coal mine workplace environment, immunofluorescence staining and qPCR were performed on colon tissues to detect the integrity of intestinal barrier and intestinal inflammatory level in model mice. There was no significant decrease in the levels of tight junction proteins ZO-1 and Occludin in CEBS mice. However, there was an over-activation of the immune response with a significant increase in MPO^+^ and CD11b^+^. Infiltration of neutrophils and macrophages has been recognized as a marker of colitis (Lei et al., 2021; Schiechl et al., 2011). Therefore, we hypothesized that stress in the coal mine workplace environment can induce severe colonic injury, the mechanical barrier is not damaged in mice. As a result of changes in the expression of tight junction proteins, the intestinal epithelium is disrupted, allowing more bacteria to cross the barrier and activating macrophages to produce TNF, IL-12, IL-23, and IL-6, leading to an inflammatory response (Catherine et al., 2023). TNF-α, IL-1b, and IL-6 have been shown to promote colonic inflammation (Younghoon et al., 2015; Marafini et al., 2019), with TNF-α causing damage to the intestinal mechanical barrier (Horiuchi et al., 2010), and IL-10 and IL-12, which are anti-inflammatory cytokines (Desbonnet et al., 2014; Lobionda et al., 2019), which can inhibit inflammation. The experimental results revealed that the CEBS mice exhibited significant inflammatory features in the levels of IL-1b, IL-6, and IL-12. These findings were consistent with the previously presented H&E and immunofluorescence staining results, which showed no significant damage to the intestinal mechanical barrier but did indicate an obvious inflammatory response. In animal models of colitis in mice, significant damage to the intestinal barrier was always accompanied by an inflammatory response, which showed discrepancies with the characteristics shown in the current study results. However, it is worth noting that some scholars have studied the intestinal pathological manifestations in a mouse model of depression. The results revealed that the mouse also exhibited elevated levels of intestinal inflammation without significant damage to the intestinal mechanical barrier (Song et al., 2022). Intestinal dysfunction caused by the coal mine workplace environment may have a similar pathological mechanism.

Based on the analysis of intestinal pathology caused by the coal mine workplace environment, we conducted 16S rRNA sequencing on the feces of CEBS mice. The results of the alpha and beta diversity analyses revealed significant differences in the gut microbiota of the CEBS mice compared to the control mice, with increased microbial abundance and diversity. Further analysis of gut microbial composition revealed that the coal mine workplace environment resulted in a significant decrease in the relative abundance of *Firmicutes* and *Lactobacillus*, and a significant increase in the relative abundance of *Bacteroidetes, Parabacteroides* in CEBS mice. *Firmicutes* and *Bacteroidetes* are the two main phyla that make up the gut microbiota at *Phylum*-level (Ping et al., 2022), and their proportion is relatively stable. However, disturbances in this proportion may lead to metabolic syndromes like obesity (Kadosh et al., 2020). The CEBS mice showed a significant reduction in the relative abundance of *Lactobacillus*, while microbial diversity increased significantly. This may be attributed to *Lactobacillus* is one of the major probiotics found in the intestinal tract, and it plays a crucial role in enhancing immunity, improving metabolism, and protecting against pathogenic bacteria. The significant reduction in the relative abundance of *Lactobacillus* can lead to decreased intestinal resistance to pathogens, combined with the higher number of pathogenic bacteria in the coal mine workplace environment, which led to a significant increase in the gut microbial diversity of CEBS mice.

Probiotics are live microorganisms that can exert beneficial effects on host health and have been shown to be effective in improving colonic inflammation (Kaur et al., 2020). Strains of *Lactobacillus* and *Bifidobacteria* are frequently used as probiotics, which have various physiological effects such as immune regulation and metabolism (Long-Smith et al., 2020). Lactobacillus Royale (L.PB-LR09), Lactobacillus suisse (PB-LR76), Lactobacillus rhamnosus (HH-LA26), and Lactobacillus acidophilus (HH-LH17) have been shown to attenuate inflammatory responses, restore intestinal barriers, and improve intestinal health in relevant studies (Lobionda et al., 2019). Lactobacillus Royale has a strong colonization ability in the lower pH environment of the gastrointestinal tract, enabling it to resist the colonization of pathogenic bacteria in the gastrointestinal tract. Multiple studies have used Lactobacillus Royale, derived from humans or other mammals, to treat mice with inflammatory bowel disease. This probiotic can regulate intestinal immune cells, alleviate inflammatory responses, reduce the levels of pro-inflammatory factors such as TNF-α and IL-12, and alleviate symptoms of diarrhea and constipation. Similarly, Lactobacillus suisse, Lactobacillus rhamnosus, and Lactobacillus acidophilus have demonstrated comparable effectiveness in individual probiotic treatments. Therefore, in this work, these four *Lactobacillus* species were compounded into a probiotic agent to intervene in CEBS mice. The intestinal function of the CEBS mice was improved, tissue morphology was restored, and intestinal inflammatory response was significantly reduced after supplementation with the compound probiotics. These results are consistent with previous studies, suggesting a significant correlation between intestinal dysfunction caused by the coal mine workplace environment and the disturbance of gut microbiota.

Overall, the coal mine workplace environment has disrupted the gut microbiome in mice, leading to intestinal dysfunction. Supplementing compound probiotics is a potential means of intervening in intestinal dysfunction caused by the coal mine workplace environment. These findings provide valuable insights into the pathogenesis of intestinal diseases in coal miners and inform potential intervention strategies. The experimental research conducted in this paper is based on animal model. Although there is a significant genomic similarity between mice and humans, some discrepancies may arise in the experimental results compared to human manifestations. Future research should consider collecting sufficient fecal samples from coal miners and conducting metabolomics studies, combining the findings with animal research to further explore the metabolic changes associated with the coal mine workplace environment and gut microbiota, which will help enhance the accuracy of animal trial results.

## 5 Conclusion

In this paper, we focus on studying the intestinal issues faced by coal miners and how the coal mine workplace environment affects the gut microbiota. We also explore intervention methods for addressing these intestinal problems. After exposing CEBS mice to the coal mine workplace environment for 4 weeks, we observed that the mice developed diarrhea and increased levels of intestinal inflammation, while the integrity of the intestinal mechanical barrier remained unaffected. Our analysis of the diversity of intestinal microbes indicates that the coal mine workplace environment plays a significant role in disrupting the balance of intestinal microbial homeostasis in coal miners, leading to intestinal dysfunction. Furthermore, we found that administering compound probiotics to mice in the coal mine workplace environment improved their colonic inflammation.

## Data Availability

The data presented in the study are deposited in the NCBI Sequence Read Archive repository, accession number PRJNA1190071: https://www.ncbi.nlm.nih.gov/bioproject/PRJNA1190071.

## References

[B1] Adolph T. E. Zhang J. W. (2022). Diet fuelling inflammatory bowel diseases: preclinical and clinical concepts. Gut 71, 2574–2586. 10.1136/gutjnl-2021-326575 36113981 PMC9664119

[B2] Arnesen H. Hitch T. C. A. Steppeler C. Bjørge M. M. H. Emilie K. L. Gjermund G.. (2021). Naturalizing laboratory mice by housing in a farmyard-type habitat confers protection against colorectal carcinogenesis. Gut Microbes 13:1993581. 10.1080/19490976.2021.1993581 34751603 PMC8583187

[B3] Barlow J. T. Leite G. Romano A. E. Sedighi R. Chang C. Celly S.. (2021). Quantitative sequencing clarifies the role of disruptor taxa, oral microbiota, and strict anaerobes in the human small-intestine microbiome. Microbiome 9, 214–226. 10.1186/s40168-021-01162-2 34724979 PMC8561862

[B4] Baumgart M. Dogan B. Rishniw M. Weitzmanet G. Bosworth B. Yantissal. R.. (2007). Culture independent analysis of ileal mucosa reveals a selective increase in invasive *Escherichia coli* of novel phylogeny relative to depletion of Clostridiales in Crohn's disease involving the ileum. ISME J. 1, 403–418. 10.1038/ismej.2007.52 18043660

[B5] Black C. J. Drossman D. A. Talley N. J. Ruddy J. Ford A. C. (2020). Functional gastrointestinal disorders: advances in understanding and management. Lancet 396, 1664–1674. 10.1016/S0140-6736(20)32115-2 33049221

[B6] Black C. J. Ford A. C. (2020). Global burden of irritable bowel syndrome: trends, predictions and risk factors. Nat. Rev. Gastroenterol. Hepatol. 17, 473–486. 10.1038/s41575-020-0286-8 32296140

[B7] Bolyen E. Rideout J. R. Dillon M. R. Bokulich N. A. Abnet C. C. Al-Ghalith G. A.. (2019). Reproducible, interactive, scalable and extensible microbiome data science using QIIME 2. Nat. Biotechnol. 37, 852–857. 10.1038/s41587-019-0209-9 31341288 PMC7015180

[B8] Catherine L. B. Sailish H. Laurent P. (2023). Ulcerative colitis. Lancet 402, 571–584. 10.1016/S0140-6736(23)00966-2 37573077

[B9] Desbonnet L. Clarke G. Shanahan F. Dinan T. G. Cryan J. F. (2014). Microbiota is essential for social development in the mouse. Mol. Psychiatry 19, 146–158. 10.1038/mp.2013.65 23689536 PMC3903109

[B10] Frank D. N. Stamand A. L. Feldman R. A. Pace N. R. (2007). Molecular-phylogenetic characterization of microbial community imbalances in human inflammatory bowel diseases. Proc. Natl. Acad. Sci. USA. 104, 13780–13785. 10.1073/pnas.0706625104 17699621 PMC1959459

[B11] Horiuchi T. Mitoma H. Harashima S. Tsukamoto H. Shimoda T. (2010). Transmembrane TNF-alpha: structure, function and interaction with anti-TNF agents. Rheumatology 49, 1215–1228. 10.1093/rheumatology/keq031 20194223 PMC2886310

[B12] Ijaz M. Akram M. Ahmad R. S. Mirza K. Nadeem F. A. Thygerson S. M.. (2020). Risk factors associated with the prevalence of upper and lower back pain in male underground coal miners in Punjab, Pakistan. Int. J. Environ. Res. Public Health 17:4102. 10.3390/ijerph17114102 32526830 PMC7312123

[B13] Jafarnejad S. Shab B. S. Speakman J. R. Parastui K. Daneshi-Maskooni M. Djafarian K.. (2016). Probiotics reduce the risk of antibiotic-associated diarrhea in adults (18-64 years) but not the elderly (>65 Years): a meta-analysis. Nutr. Clin. Pract. 31, 502–513. 10.1177/0884533616639399 27130655

[B14] Jang Y. J. Kim W. K. Han D. H. Lee K. Ko G. (2019). *Lactobacillus fermentum* species ameliorate dextran sulfate sodium-induced colitis by regulating the immune response and altering gut microbiota. Gut Microbes 10, 696–711. 10.1080/19490976.2019.1589281 30939976 PMC6866707

[B15] Kadosh E. Snir-Alkalay I. Venkatachalam A. May S. Lasry A. Elyada E.. (2020). The gut microbiome switches mutant p53 from tumour-suppressive to oncogenic. Nature 586, 133–156. 10.1038/s41586-020-2541-0 32728212 PMC7116712

[B16] Kanazawa M. Endo Y. Whitehead W. E. Kano M. Hongo M. Fukudo S.. (2004). Patients and nonconsulters with irritable bowel syndrome reporting a parental history of bowel problems have more impaired psychological distress. Dig. Dis. Sci. 49, 1046–1053. 10.1023/B:DDAS.0000034570.52305.10 15309899

[B17] Kassinen A. Krogius-Kurikka L. Mäkivuokko H. Rinttil T. Paulin L. Corander J.. (2007). The fecal microbiota of irritable bowel syndrome patients differs significantly from that of healthy subjects. Gastroenterology 133, 24–33. 10.1053/j.gastro.2007.04.005 17631127

[B18] Kaur L. Gordon M. Baines P. A. Iheozor-Ejiofor Z. Sinopoulou V. Akobeng A. K.. (2020). Antibiotics for the induction and maintenance of remission in ulcerative colitis. Cochrane Database Syst. Rev. 5:Cd013743. 10.1002/14651858.CD005573.pub3 32128794 PMC7059960

[B19] Kelly J. R. Borre Y. Brien C. O. Patterson E. Dinan T. G. (2016). Transferring the blues: depression-associated gut microbiota induces neurobehavioural changes in the rat. J. Psychiatr. Res. 82, 109–118. 10.1016/j.jpsychires.2016.07.019 27491067

[B20] Lei Z. Yang L. X. Lei Y. T. Yang Y. H. Zhang X. Y. Song Q.. (2021). High dose lithium chloride causes colitis through activating F4/80 positive macrophages and inhibiting expression of Pigr and Claudin-15 in the colon of mice. Toxicology 457:152799. 10.1016/j.tox.2021.152799 33901603

[B21] Levy R. L. Whitehead W. E. Von Korff M. R. Feld A. D. (2000). Intergenerational transmission of gastrointestinal illness behavior. Am. J. Gastroenterol. 95, 451–456. 10.1111/j.1572-0241.2000.01766.x 10685749

[B22] Li L. Wang S. W. Huang L. Zhi M. Cai Q. Fang Z. H.. (2022). The impacts of workplace environment on coal miners' emotion and cognition depicted in a mouse model. Front. Behav. Neurosci. 16:896545. 10.3389/fnbeh.2022.896545 35783230 PMC9245518

[B23] Li Y. D. Xiao K. Xiao S. Y. Wang M. M. Pei S. S. Liu H. L.. (2022). Difference in intestinal flora and characteristics of plasma metabonomics in pneumoconiosis patients. Metabolites 12:917. 10.3390/metabo12100917 36295819 PMC9609413

[B24] Liu J. Wang Y. X. Heelan W. J. Chen Y. Li Z. T. Hu Q. Y.. (2022). Mucoadhesive probiotic backpacks with ROS nanoscavengers enhance the bacteriotherapy for inflammatory bowel diseases. Sci. Adv. 8:eabp8798. 10.1126/sciadv.abp8798 36367930 PMC9651739

[B25] Lobionda S. Sittipo P. Kwon H. Y. Lee Y. K. (2019). The role of gut microbiota in intestinal inflammation with respect to diet and extrinsic stressors. Microorganisms. 7:271. 10.3390/microorganisms7080271 31430948 PMC6722800

[B26] Loftus E. V. (2004). Clinical epidemiology of inflammatory bowel disease: incidence, prevalence, and environmental influences. Gastroenterology 126, 1504–1517. 10.1053/j.gastro.2004.01.063 15168363

[B27] Long-Smith C. O'Riordan J. K. Clarke G. Stanton C. Dinan T. G. Cryan J. F.. (2020). Microbiota-gut-brain axis: new therapeutic opportunities. Annu. Rev. Pharmacol. Toxicol. 60, 477–502. 10.1146/annurev-pharmtox-010919-023628 31506009

[B28] Lu Y. Zhang Z. Yan H. Rui B. Liu J. (2020). Effects of occupational hazards on job stress and mental health of factory workers and miners: a propensity score analysis. Biomed Res. Int. 2020:1754897. 10.1155/2020/1754897 32904478 PMC7456464

[B29] Lu Z. H. Liu Y. W. Ji Z. H. Fu T. Yan M. Shao Z. J.. (2021). Alterations in the intestinal microbiome and mental health status of workers in an underground tunnel environment. BMC Microbiol. 21, 1–9. 10.1186/s12866-020-02056-3 33407119 PMC7788853

[B30] Lyra A. Teemu R. Janne N. Krogius-Kurikka L. Palva A. (2009). Diarrhoea-predominant irritable bowel syndrome distinguishable by 16s rRNA gene phylotype quantification. World J. Gastroenterol. 15, 5936–595. 10.3748/wjg.15.5936 20014457 PMC2795180

[B31] Maldonado-Gómez X. M. Martínez I. Bottacini F. Callaghan A. O. Ventura M. Sinderen D. V.. (2016). Stable engraftment of Bifidobacterium longum AH1206 in the human gut depends on individualized features of the resident microbiome. Cell Host Microbe. 20, 515–526. 10.1016/j.chom.2016.09.001 27693307

[B32] Mangiola F. Ianiro G. Franceschi F. Fagiuoli S. Gasbarrini G. Gasbarrini A.. (2016). Gut microbiota in autism and mood disorders. World J. Gastroenterol. 22, 361–378. 10.3748/wjg.v22.i1.361 26755882 PMC4698498

[B33] Marafini I. Sedda S. Dinallo V. Monteleone G. (2019). Inflammatory cytokines: from discoveries to therapies in IBD. Expert Opin. Biol. Ther. 19, 1207–1217. 10.1080/14712598.2019.1652267 31373244

[B34] Ni J. Wu G. D. Albenberg L. Tomov V. T. (2017). Gut microbiota and IBD: causation or correlation? Nat. Rev. Gastroenterol. Hepatol. 14, 573–584. 10.1038/nrgastro.2017.88 28743984 PMC5880536

[B35] Pan G. H. Liu B. D. Li S. X. Han M. L. Gao L. Xu G. H.. (2020). Kuijieling, A Chinese medicine alleviates DSS-induced colitis in C57BL/6J mouse by improving the diversity and function of gut microbiota. FEMS Microbiol. Lett. 367:fnaa082. 10.1093/femsle/fnaa082 32407465

[B36] Park A. J. Collins J. Blennerhassett P. A. Ghia J. E. Collins S. M. (2013). Altered colonic function and microbiota profile in a mouse model of chronic depression. Neurogastroenterol. Motil. 25:733-e575. 10.1111/nmo.12153 23773726 PMC3912902

[B37] Park S. C. Jeen Y. T. (2018). Anti-integrin therapy for inflammatory bowel disease. World J. Gastroenterol. 24, 1868–1880. 10.3748/wjg.v24.i17.1868 29740202 PMC5937204

[B38] Paz L. A. Mesquita M. Lacarte G. M. Vázquez O. E. Ubieto R. B. Gutierrez A. H.. (2024). Fecal microbiota transplantation from female donors restores gut permeability and reduces liver injury and inflammation in middle-aged male mice exposed to alcohol. Front. Nutr. 11:1393014. 10.3389/fnut.2024.1393014 38699545 PMC11063254

[B39] Ping L. L. Lei Q. Tuo L. (2022). Supplemental dietary Selenohomolanthionine affects growth and rumen bacterial population of Shaanbei white cashmere wether goats. Front. Microbiol. 13:942848. 10.3389/fmicb.2022.942848 36338028 PMC9632625

[B40] Saito Y. A. Petersen G. M. Larson J. J. Atkinson E. J. Fridley B. L. Andrade M.. (2010). Familial aggregation of irritable bowel syndrome: a family case—control study. Am. J. Gastroenterol. 105, 833–841. 10.1038/ajg.2010.116 20234344 PMC2875200

[B41] Schiechl G. Bauer B. Fuss I. Lang S. A. Moser C. Ruemmele P.. (2011). Tumor development in murine ulcerative colitis depends on MyD88 signaling of colonic F4/80+CD11b(high)Gr1(low) macrophages. J. Clin. Invest. 121, 1692–1708. 10.1172/JCI42540 21519141 PMC3083803

[B42] Seethaler B. Nguyen N. K. Basrai M. Kiechle M. Walter J. Delzenne N. M.. (2022). Short-chain fatty acids are key mediators of the favorable effects of the Mediterranean diet on intestinal barrier integrity: data from the randomized controlled LIBRE trial. Am. J. Clin. Nutr. 116, 928–942. 10.1093/ajcn/nqac175 36055959

[B43] Seksik P. Rigottier-Gois L. Gramet G. Sutren M. Pochart P. Marteau P.. (2003). Alterations of the dominant faecal bacterial groups in patients with Crohn's disease of the colon. Gut 52, 237–242. 10.1136/gut.52.2.237 12524406 PMC1774977

[B44] Shalini E. Borody J. T. Damian M. R. H. (2022). Fecal microbiota transplantation reduces pathology and improves cognition in a mouse model of alzheimer's disease. Cells 12, 119–119. 10.3390/cells12010119 36611911 PMC9818266

[B45] Sokol H. Pigneur B. Watterlot L. Lakhdari O. Bermúdez-Humarán L. G. Gratadoux J.. (2008). *Faecalibacterium prausnitzii* is an anti-inflammatory commensal bacterium identified by gut microbiota analysis of Crohn disease patients. Proc. Natl. Acad. Sci. USA. 105, 16731–16736. 10.1073/pnas.0804812105 18936492 PMC2575488

[B46] Song X. J. Wang W. H. Ding S. S. Wang Y. Ye L. F. Chen X.. (2022). Exploring the potential antidepressant mechanisms of puerarin: anti-inflammatory response via the gut-brain axis. J. Affect. Disord. 310, 459–471. 10.1016/j.jad.2022.05.044 35568321

[B47] Sperber A. D. Bangdiwala S. I. Drossman D. A. Ghoshal U. C. Simren M. Tack J.. (2021). Worldwide prevalence and burden of functional gastrointestinal disorders, results of Rome Foundation Global Study. Gastroenterology 160, 99–114. 10.1053/j.gastro.2020.04.014 32294476

[B48] Sperber A. D. Dan D. Fukudo S. Gerson C. Ghoshal U. C. Gwee K. A.. (2017). The global prevalence of IBS in adults remains elusive due to the heterogeneity of studies: a Rome Foundation working team literature review. Gut 66, 1075–1082. 10.1136/gutjnl-2015-311240 26818616

[B49] Wang X. W. Xing Y. T. Ji Y. L. Xi Liu H. Y. Yang X. H. L.. (2022). The Combination of phages and faecal microbiota transplantation can effectively treat mouse colitis caused by *Salmonella enterica* Serovar Typhimurium. Front. Microbiol. 13, 944495. 10.3389/fmicb.2022.944495 35875536 PMC9301289

[B50] Whitehead W. E. Palsson O. Jones K. R. (2002). Systematic review of the comorbidity of irritable bowel syndrome with other disorders: what are the causes and implications? Gastroenterology 122, 1140–1156. 10.1053/gast.2002.32392 11910364

[B51] Whorwell P. J. Mccallum M. Creed F. H. Roberts C. T. (1986). Non-colonic features of irritable bowel syndrome. Gut 27, 37–40. 10.1136/gut.27.1.37 3949235 PMC1433171

[B52] Wu J. C. (2011). Community-based study on psychological comorbidity in functional gastrointestinal disorder. J. Gastroenterol. Hepatol. 26, 23–26. 10.1111/j.1440-1746.2011.06642.x 21443703

[B53] Xie H. P. Liu J. F. Gao M. Z. Liu Y. L. Li W. M. (2020). Physical symptoms and mental health status in deep underground miners: a cross-sectional study. Medicine 99:e19294. 10.1097/MD.0000000000019294 32118742 PMC7478699

[B54] Xu L. M. Liu B. D. Huang L. J. Li Z. Cheng Y. B. Tian Y.. (2022). Probiotic consortia and their metabolites ameliorate the symptoms of inflammatory bowel diseases in a colitis mouse model. Microbiol. Spectr. 10:e0065722. 10.1128/spectrum.00657-22 35730951 PMC9430814

[B55] Yang Z. W. Chen Z. Lin X. L. Yao S. Y. Xian M. Ning X. P.. (2022). Rural environment reduces allergic inflammation by modulating the gut microbiota. Gut Microbes 14:2125733. 10.1080/19490976.2022.2125733 36193874 PMC9542937

[B56] Yilmaz B. Juillerat P. Oyas O. Ramon C. Bravo F. D. Franc Y.. (2019). Microbial network disturbances in relapsing refractory Crohn's disease. Nat. Med. 25, 323–336. 10.1038/s41591-018-0308-z 30664783

[B57] Yin H. M. Zhong Y. D. Wang H. Hu J. L. Xia S. K. Xiao Y. D.. (2022). Short-term exposure to high relative humidity increases blood urea and influences colonic urea-nitrogen metabolism by altering the gut microbiota. J. Adv. Res. 35, 153–168. 10.1016/j.jare.2021.03.004 35003799 PMC8721250

[B58] Younghoon K. Hoon J. K. Jun A. Y. Sejong O. Hun K. S. (2015). The synergic anti-inflammatory impact of *Gleditsia sinensis* Lam., *Lactobacillus brevis* KY21 on intestinal epithelial cells in a DSS-induced colitis model. Korean J. Food Sci. Anim. Resour. 35, 604–610. 10.5851/kosfa.2015.35.5.604 26761887 PMC4670888

[B59] Zahraa B. A. Dekker M. N. Aya M. Negar N. (2020). The gut microbiota and inflammation: an overview. Int. J. Environ. Res. Public Health 17:7618. 10.3390/ijerph17207618 33086688 PMC7589951

[B60] Zoetendal E. G. Rajilic-Stojanovic M. de Vos W. M. (2008). High-throughput diversity and functionality analysis of the gastrointestinal tract microbiota. Gut 57, 1605–1615. 10.1136/gut.2007.133603 18941009

