# Supplementary material for: The effect of workplace environment on coal miners' gut microbiota in a mouse model
Source: Front Microbiol. 2024 Dec 11;15:1453798. doi: 10.3389/fmicb.2024.1453798 (PMC11668784; doi:10.3389/fmicb.2024.1453798)
Supplement: Supplementary file 2 [file Supplementary_file_2.docx]

Supplementary Material

# Supplementary Figures


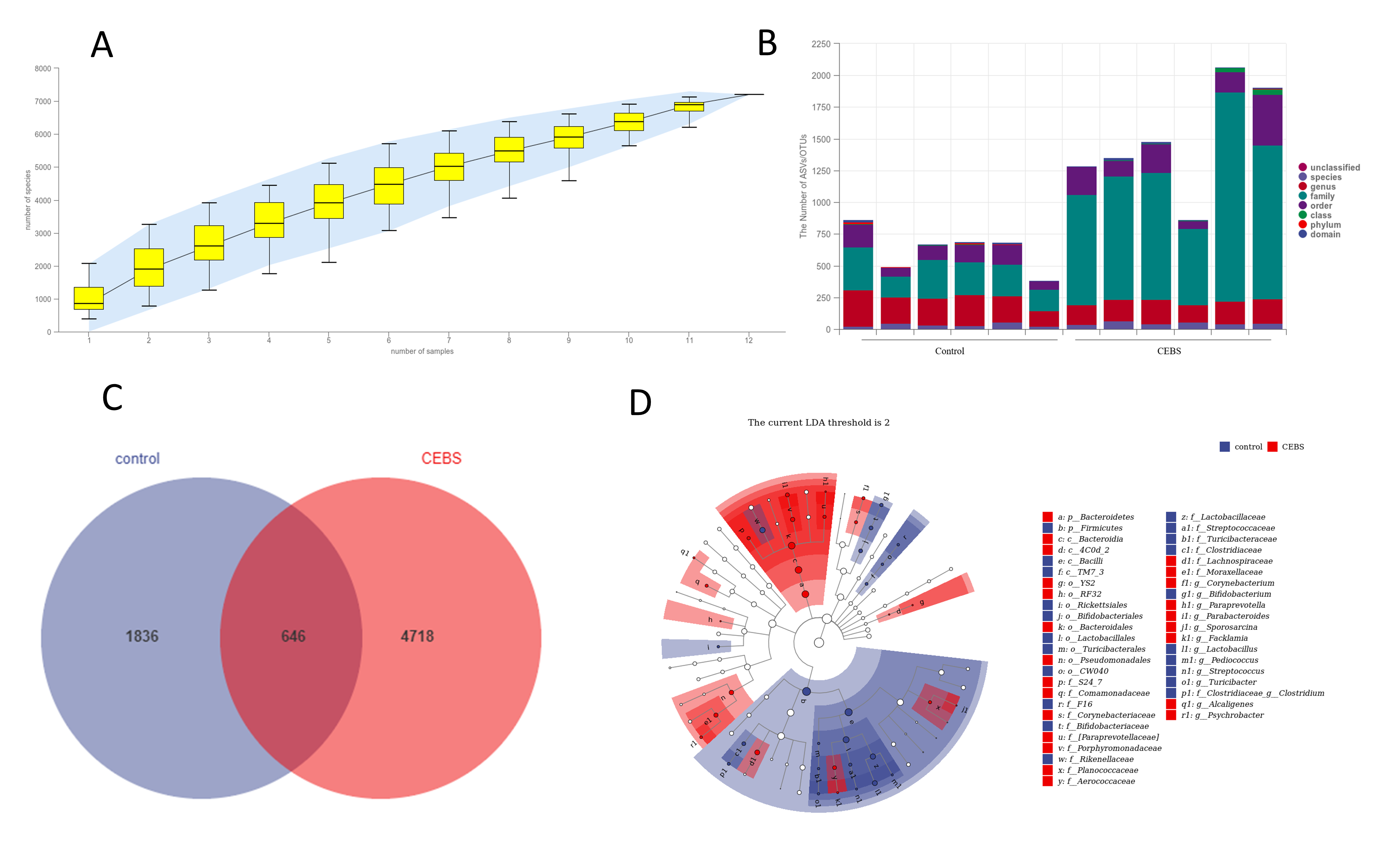


**Supplementary Figure 1.** **Classification of gut microbia species.** (A) Species cumulative curves. (B) Taxonomic annotation of species. (C) ASV abundance. (D) Evolutionary branching diagram for LEfSe analysis.


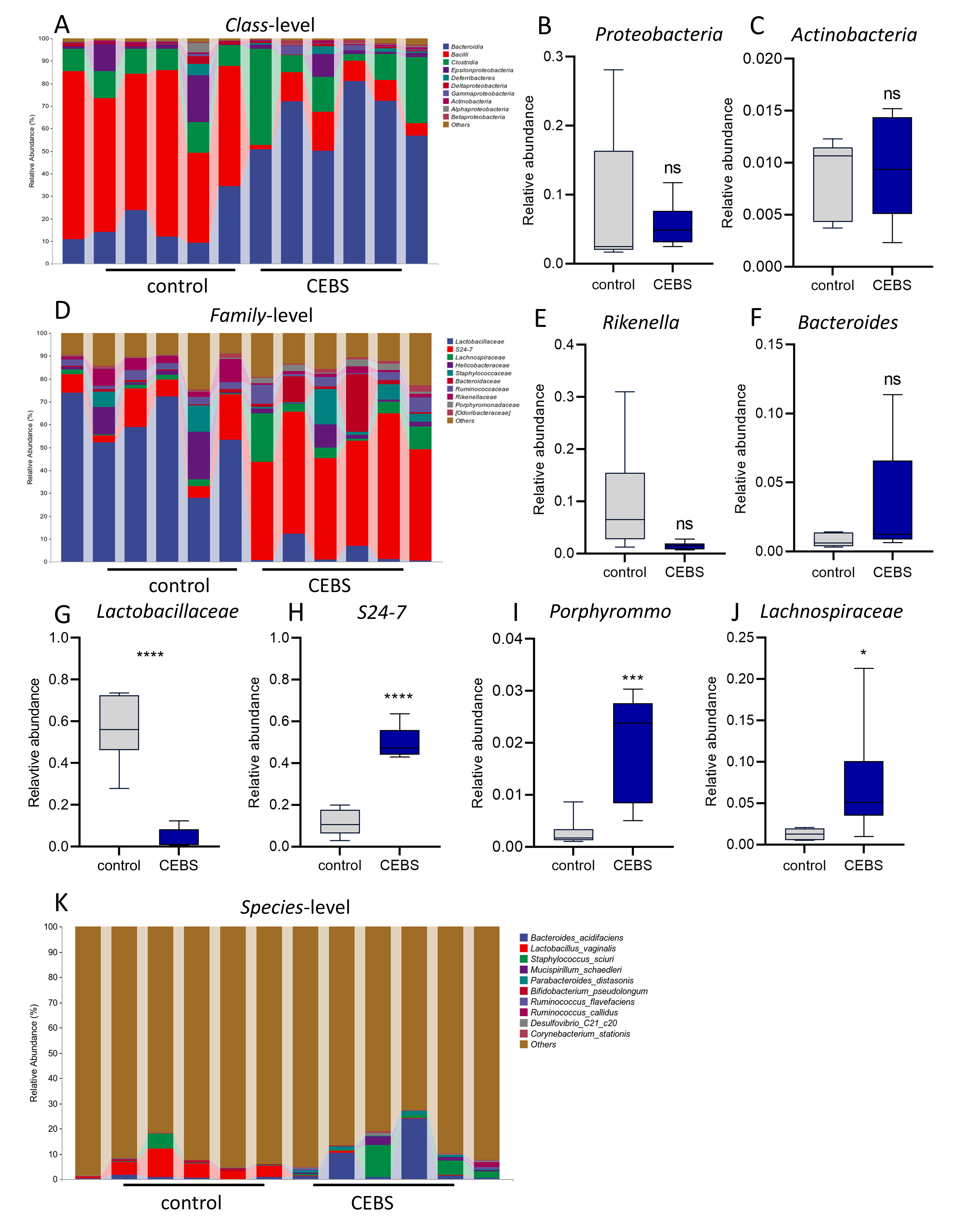


**Supplementary Figure 2.** **Analysis of gut microbiota composition at different levels.** (A) The relative abundance of gut microbiota at Class-level. (B) The relative abundance of Proteobacteria. (C) The relative abundance of Actinobacteria. (D) The relative abundance of gut microbiota at Family-level. (E) The relative abundance of Rikenella. (F) The relative abundance of Bacteroides. (G) The relative abundance of Lactobacillaceae. (H) The relative abundance of S24-7. (I) The relative abundance of Porphyrommo. (J) The relative abundance of Lachnospiraceae. (K) The relative abundance of gut microbiota at Species-level. (control mice vs. CEBS mice). ns, *P < 0.05, ***P < 0.001, and ****P < 0.0001.


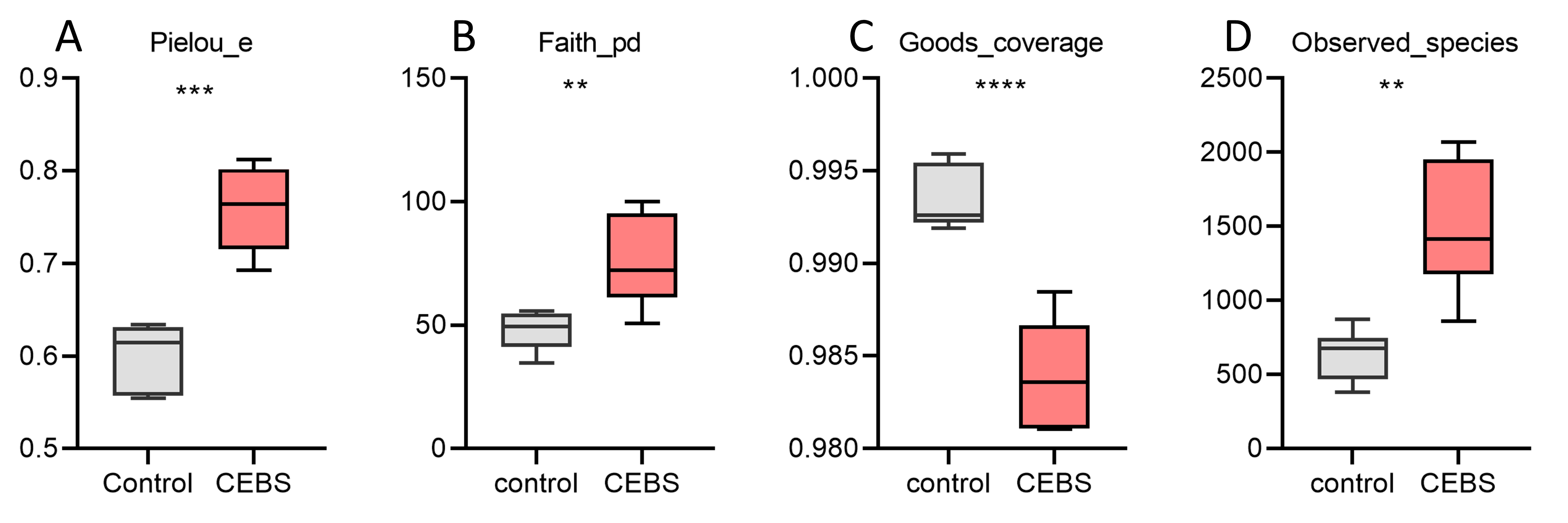


**Supplementary Figure 3.** **Alpha diversity analysis.** (A) Pielou_e. (B) Faith_pd. (C) Goods_coverage. (D) Observed_species. (control mice vs. CEBS mice). **P < 0.01, and ****P < 0.0001.
